# Notch Inhibition Prevents Differentiation of Human Limbal Stem/Progenitor Cells *in vitro*

**DOI:** 10.1038/s41598-019-46793-6

**Published:** 2019-07-17

**Authors:** Sheyla González, Heui Uhm, Sophie X. Deng

**Affiliations:** 1 Cornea Division, Stein Eye Institute, University of California, Los Angeles, CA 90095 USA; 2 David Geffen School of Medicine, University of California, Los Angeles, CA 90095 USA

**Keywords:** Cell signalling, Adult stem cells

## Abstract

Notch signaling has been shown to regulate the homeostasis and wound healing of the corneal epithelium. We investigated the effect of Notch inhibition in the human limbal stem/progenitor cells (LSCs) *in vitro* by using small molecules. Treatment of the LSCs with DAPT and SAHM1 reduced the proliferation rate and maintained the undifferentiated state of the LSCs in a concentration dependent manner. Stratification and differentiation of the corneal epithelium were not reduced after Notch inhibition, indicating that the function of the corneal basal cells is retained. Our findings suggest that Notch signaling plays a role in the proliferation and maintenance of LSCs.

## Introduction

Maintenance of the integrity of the corneal epithelium is crucial for preserving its refractive power and its barrier function against environmental insults. The corneal epithelium is regenerated by a population of adult stem cells, the limbal epithelial stem/progenitor cells (LSCs), that reside at the sclerocorneal junction between the conjunctiva and cornea called limbus^1–4^. Limbal stem cell deficiency (LSCD) is a condition characterized by the loss or dysfunction of LSCs. Understanding the regulation of the LSCs is crucial for the development of new treatments for patients suffering from LSCD.

Approaches to maintain the undifferentiated state of LSCs *ex vivo* are focused on emulating the *in vivo* microenvironment. Multiple signals are involved in the maintenance of the corneal homeostasis and wound healing^5,6^. The specific molecular mechanisms underlying self-renewal, proliferation, stratification and differentiation of the corneal epithelium are still poorly understood, albeit an extensive body of literature has been published.

Notch signaling is a developmentally conserved signaling pathway that functions in different embryonic stages and multiple adult cell types, and controls multiple cell processes such as proliferation, differentiation, cell fate and cell death. Although Notch signaling has a very simple design in terms of the number of proteins involved in the pathway, it is a very complex and versatile pathway based on the different cell responses it can trigger^7,8^. Activation of Notch signaling by binding of the ligand to the Notch receptors promotes the transcription of Notch-dependent target genes such as *HES1* (hairy and enhancer of split-1), *HES5* (hairy and enhancer of split-5) and *HEY1* (hairy/enhancer-of-split related with YRPW motif protein 1).

The expression of several molecules involved in the Notch signaling have been reported in the human cornea^9–13^. Notch1 and HES1 have been detected at the basal layer of the limbal epithelium where LSCs are located^10–12^.

During development, Notch signaling has been proposed to maintain the LSC phenotype in mice^12^. Inhibition of Notch has been shown to reduce the amount of LSC proliferating cells and increase the amount of differentiated cells^9^. Opposite results were found when activation with a recombinant jagged1 protein was done^9^.

Notch signaling appears to play an important role in epithelial wound healing. Cornea wound healing has been shown to induce Notch signaling and increase proliferation in the Notch1 intracellular domain (N1IC) transgenic mice^14^. Notch1 has also been proposed to play an important role in the corneal epithelial barrier function^15^.

However, the role of Notch signaling in human LSCs is not well understood. In this study, we investigated the role of Notch signaling in human LSCs using two small Notch inhibitors, DAPT and SAHM1. DAPT inhibits Notch signaling by targeting gamma secretase, an integral membrane protein that cleaves Notch receptors^16^; SAHM1 inhibits Notch signaling by preventing the assembly of the transcription complex^17^.

## Results

### Notch signaling is present at the basal layer of the human limbal epithelium

**Figure 1:**
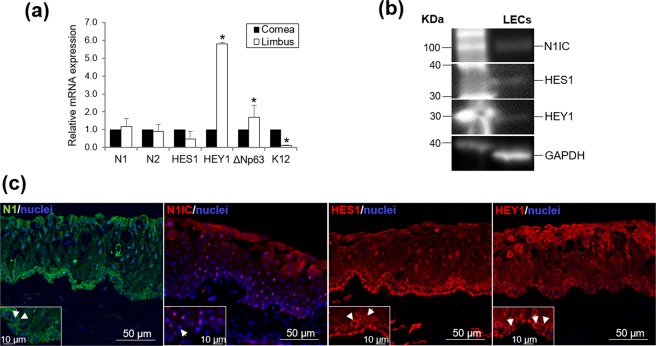
Expression of the Notch family members in the human cornea. **(a)** Expression of N1 and N2 receptors, and HES1 and HEY1 target genes at the mRNA level by qRT-PCR. ΔNp63 and K12 were used as positive controls to determine the expression level in the cornea *versus* the limbus. **(b)** Expression of N1IC, HES1 and HEY1 in the LECs at the protein level by Western Blot; GAPDH was the housekeeping gene used. **(c)** Expression of N1, N1IC, HES1 and HEY1 at the protein level by IHC on cross-sections of sclerocorneal tissue. Arrows in the inserts indicate expression in the nuclei at the basal layer. Arrowheads in the inserts indicate expression at the membrane/cytoplasm. Data are represented as mean ± SEM. See also Fig. S3. Abbreviations: GAPDH, Glyceraldehyde-3-Phosphate Dehydrogenase; HES1, hairy and enhancer of split-1; HEY1, hairy/enhancer-of-split related with YRPW motif protein 1; IHC, immunohistochemistry; K12, cytokeratin 12; LECs, Limbal Epithelial Cells; N1, Notch1; N2, Notch2; N1IC, Notch1 intracellular domain.

At the mRNA level, Notch1 (N1), Notch2 (N2) and HES1 were expressed in the epithelium at a similar level in both the central cornea and limbus; instead, HEY1 showed a higher expression in the limbus (Fig. 1A). As expected, cytokeratin (K) 12 had a lower expression in the limbus and ΔNp63 had a higher expression in the limbus. These two markers were used as controls.

At the protein level, the expression of the Notch1 intracellular domain (N1IC), HES1 and HEY1 was detected in primary human limbal epithelial cells by Western blot (Fig. 1B and Fig. S3). N1, HEY1 and HES1 were predominantly detected in the basal limbal epithelium and at a lesser degree at the superficial layer (Fig. 1C) by immunohistochemistry. N1 was mainly detected at the membrane of the limbal basal cells (arrowhead in the insert of Fig. 1C, N1); however, some foci-like staining was also seen in the nuclei (arrow in the insert of Fig. 1C, N1). HEY1 and HES1 were mainly detected in the cytoplasm of the basal cells (arrowhead in the insert of Fig. 1C); some foci-like staining was also seen in the nuclei (arrow in the insert of Fig. 1C, HEY1 and HES1).

N1IC was detected in the basal and suprabasal limbal epithelial layer (Fig. 1C, N1IC); strong foci-like staining was seen in the nuclei of the cells at the basal and suprabasal layers of the limbus (arrow in the insert of Fig. 1C, N1IC).

### DAPT and SAHM1 inhibit Notch signaling in the limbal epithelial cells

The inhibition of Notch signaling in the freshly isolated primary limbal epithelial cells was assessed after 24 hours of the incubation with DAPT or SAHM1 at different concentrations by analyzing (1) the expression of Notch target genes HES1, HES5 and HEY1 at the mRNA level and (2) expression and cellular localization of the N1IC at the protein level. LN1-7 cells expressing N1 ectopically were used as the positive control. This cell line has been previously used to study Notch signaling^18^.

**Figure 2:**
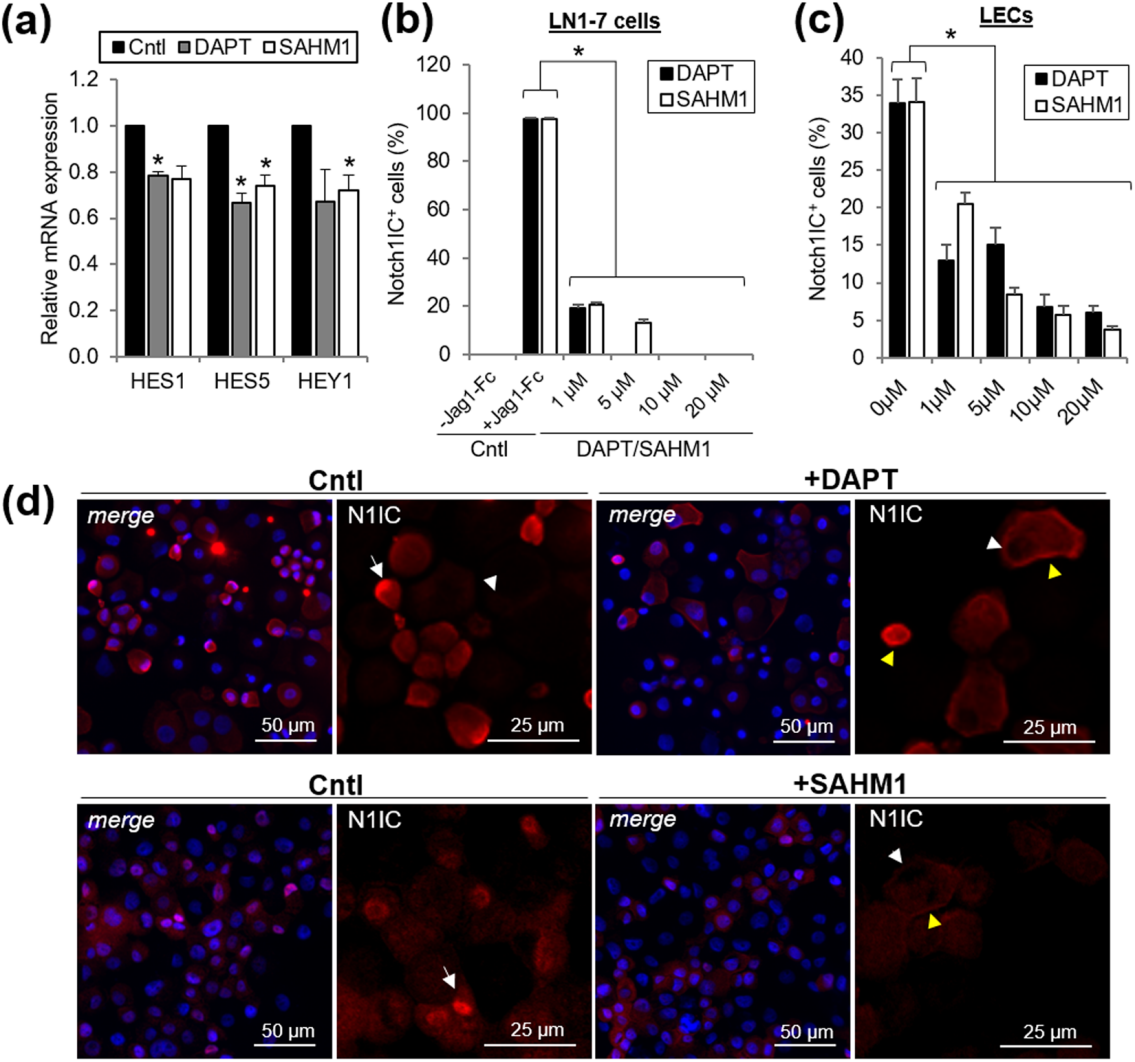
Inhibition of Notch signaling by DAPT and SAHM1 in the LECs for 24 hours. **(a)** Relative mRNA expression of Notch target genes HES1, HES5 and HEY1 in the LECs with DAPT and SAHM1. **(b)** Percentage of nuclear N1IC^+^ cells in the control LN1-7 cell line after incubation with Jag1-Fc (positive control) and in presence of different concentrations of DAPT/SAHM1. **(c)** Percentage of nuclear N1IC^+^ cells in the LECs in basal conditions (0 µM, control) and in presence of different concentrations of DAPT/SAHM1. **(d)** Representative images of N1IC immunostaining in the LECs in the presence of DAPT and SAHM1. White arrow: localization of N1IC in the nucleus (Notch activation); white arrowhead: absence of N1IC in the nucleus (Notch inhibition); yellow arrowhead: N1IC associated to the cell membrane (Notch inhibition). Data are represented as mean ± SEM. Abbreviations: Cntl, Control; HES1, hairy and enhancer of split-1; HES5, hairy and enhancer of split-5; HEY1, hairy/enhancer-of-split related with YRPW motif protein 1; LECs, limbal epithelial cells; N1IC, Notch1 intracellular domain;. *donates *p* < 0.05.

DAPT at 1 μM reduced the mRNA expression of HES1 (*P* = 0.01) and HES5 (*P* = 0.02; Fig. 2A). SAHM1 at 1 μM reduced the expression of HES5 (*P* = 0.02) and HEY1 (*P* = 0.04; Fig. 2A).

At the protein level, the expression of nuclear N1IC in the control LN1-7 cell line decreased in the presence of DAPT or SAHM1 at all the concentrations tested (*P* < 0.05; Fig. 2B).

Nuclear N1IC was detected in 34% ± 3.1% of the limbal epithelial cells that were not treated (Fig. 2C,D). When treated with DAPT or SAHM1, the expression of nuclear N1IC was significantly reduced at all concentrations tested (*P* < 0.05; Fig. 2C,D). These findings showed that DAPT and SAHM1 could inhibit Notch signaling in the limbal epithelial cells.

### Notch inhibition by DAPT and SAHM1 maintained the LSC phenotype

We next examined whether inhibition of Notch signaling using DAPT and SAHM1 had any effect on the LSC phenotype. The undifferentiated state of the treated LSCs was evaluated by assessing cell morphology, the colony forming efficiency (CFE), and by analyzing the expression of putative stem cell markers and the population of small cells.

**Figure 3:**
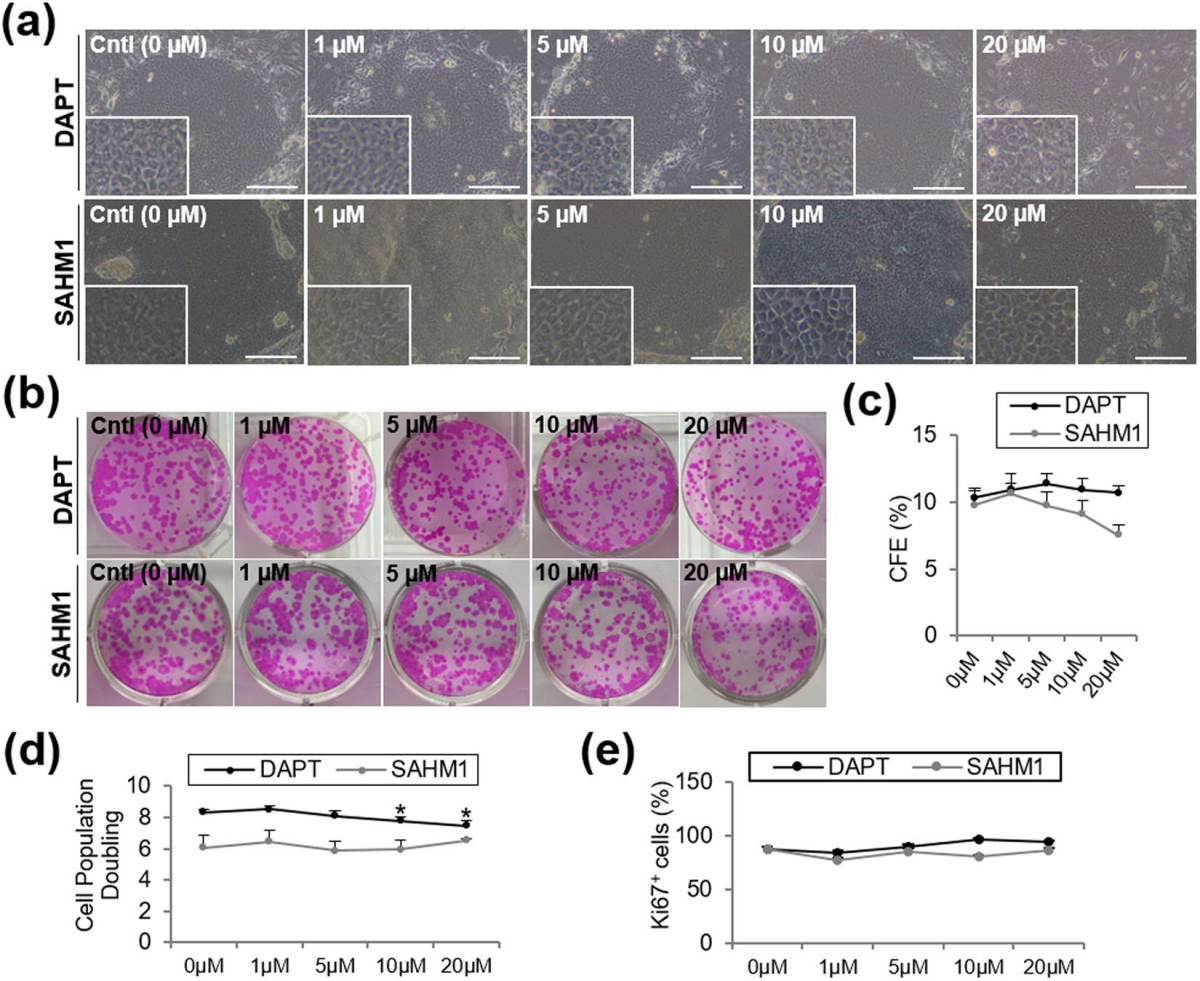
Growth of LSCs in the presence of DAPT and SAHM1 at different concentrations. **(a)** Morphology of LSC colonies with DAPT and SAHM1 at different concentrations. Insert shows morphology of cells in the colony at a higher magnification. Scale bar: 100 µm. **(b)** Representative images of Rhodamine B staining of the LSC colonies. **(c)** CFE quantification on the Rhodamine B-stained plates. **(d)** Cell population doubling of the LSCs with DAPT/SAHM1. **(e)** Quantification of Ki67^+^ cells at the protein level by ICC. Data are represented as mean ± SEM. Abbreviations: CFE, colony forming efficiency; Cntl, Control; ICC, immunocytochemistry; LSC: limbal stem cells.

Cultivated LSCs in the presence of DAPT or SAHM1 showed very similar cell morphology to those in the absence of Notch inhibition (Fig. 3A). CFE in the presence of DAPT and SAHM1 was comparable to the control in all conditions tested (*P* > 0.05; Fig. 3B,C). However, the colony size decreased in high concentrations of DAPT and SAHM1 (Fig. 3A,B).

**Figure 4:**
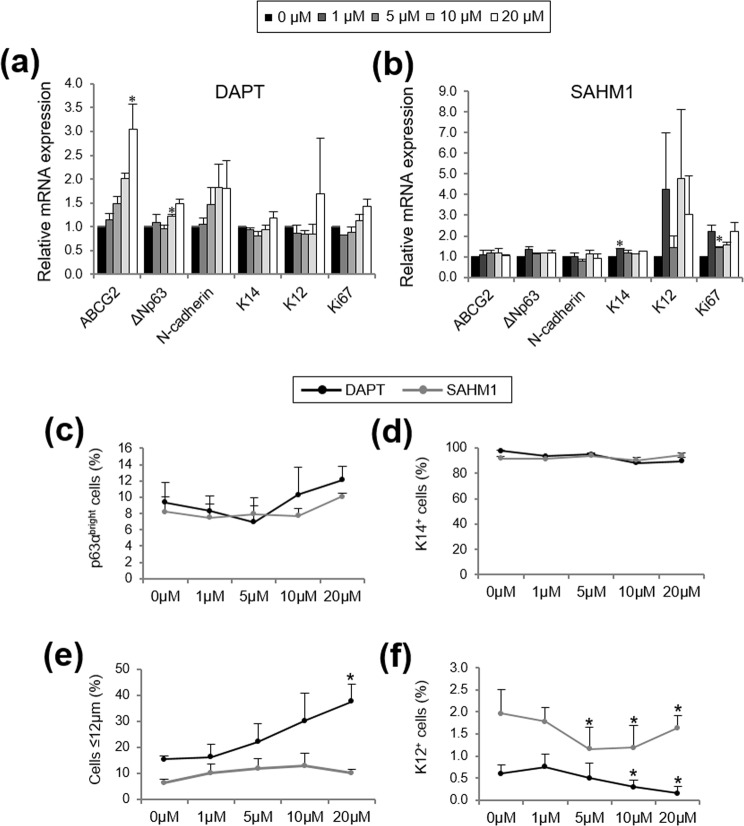
Characterization of LSCs cultured with DAPT and SAHM1. **(a)** Markers’ expression at the mRNA level in the presence of different concentrations of DAPT. **(b)** Markers’ expression at the mRNA level in the presence of different concentrations of SAHM1. **(c)** Quantification of p63α^bright^ cells at the protein level by ICC. **(d)** Quantification of K14^+^ cells at the protein level by ICC. **(e)** Percentage of small (≤12 µm) LSC-like cells in the LSC cultures. **(f)** Quantification of K12^+^ cells at the protein level by ICC. Data are represented as mean ± SEM. See also Fig. S2. Abbreviations: ABCG2, ATP Binding Cassette Subfamily G Member 2; ICC, immunocytochemistry; K12, cytokeratin 12; K14, cytokeratin 14; LSCs, limbal stem/progenitor cells.

Increasing concentrations of DAPT generated a progressive increase in the mRNA expression of ABCG2, ΔNp63, N-cadherin and K14 (Fig. 4A); however only the expression of ABCG2 at 20 μM (*P* = 0.02) and ΔNp63 at 10 μM (*P* = 0.03) reached statistical significance. K12 mRNA levels did not change when Notch signaling was inhibited (Fig. 4A). In the presence of SAHM1, there was no difference in the mRNA expression of ABCG2, ΔNp63, N-cadherin and K14 except for K14 at 1 μM (*P* = 0.02; Fig. 4B). The expression of K12 was variable (*P* > 0.05; Fig. 4B).

At the protein level, quantification of the percentage of p63α high-expressing cells (p63α^bright^ cells) revealed no statistically significant differences in the presence of either DAPT or SAHM1 at any of the concentrations tested (range, 7.0% to 12.1%, *P* > 0.05; Fig. 4C). However, a trend towards increasing percentage of p63α^bright^ cells with increasing concentrations of both DAPT and SAHM1 was observed (Fig. 4C). A high percentage of undifferentiated K14^+^ cells was found in all the conditions tested for both DAPT and SAHM1 (range, 88.2% to 97.6%; all *P* > 0.05; Fig. 4D). A progressive increase of the number of small cells (≤12 μm) was observed in the cultivated LSCs with both DAPT and SAHM1 (Fig. 4E); DAPT at 20 μM showed significant differences (*P* < 0.05; Fig. 4E). The percentage of differentiated K12^+^ cells decreased at high concentrations of DAPT and SAHM1 (10–20 µM DAPT and 5–20 µM SAHM1, *P* < 0.05; Fig. 4F).

### DAPT and SAHM1 decreased the LSC proliferation rate

There was a progressive reduction in the cell population doubling with increasing concentrations of DAPT and SAHM1 (Fig. 3D); significant differences were found for 10 and 20 μM DAPT (*P* < 0.05; Fig. 3D).

There was a large percentage of Ki67^+^ cells in culture which suggests that a large amount of cells still have the capacity to proliferate in all the conditions tested (Fig. 3E). However, no significant differences were found among the conditions tested (*P* > 0.05; Fig. 3E).

Ki67 mRNA expression did not change after DAPT treatment (*P* > 0.05; Fig. 4A). However, SAHM1 treatment increased Ki67 mRNA expression at 5 µM (*P* = 0.04; Fig. 4B).

Because Ki67 protein is present during all active phases of the cell cycle except G0 (quiescent cells), Ki67 labels cells that retain the capacity to proliferate; EdU incorporation, instead, determines the percentage of actively dividing cells (S-phase) at a specific time point.

**Figure 5:**
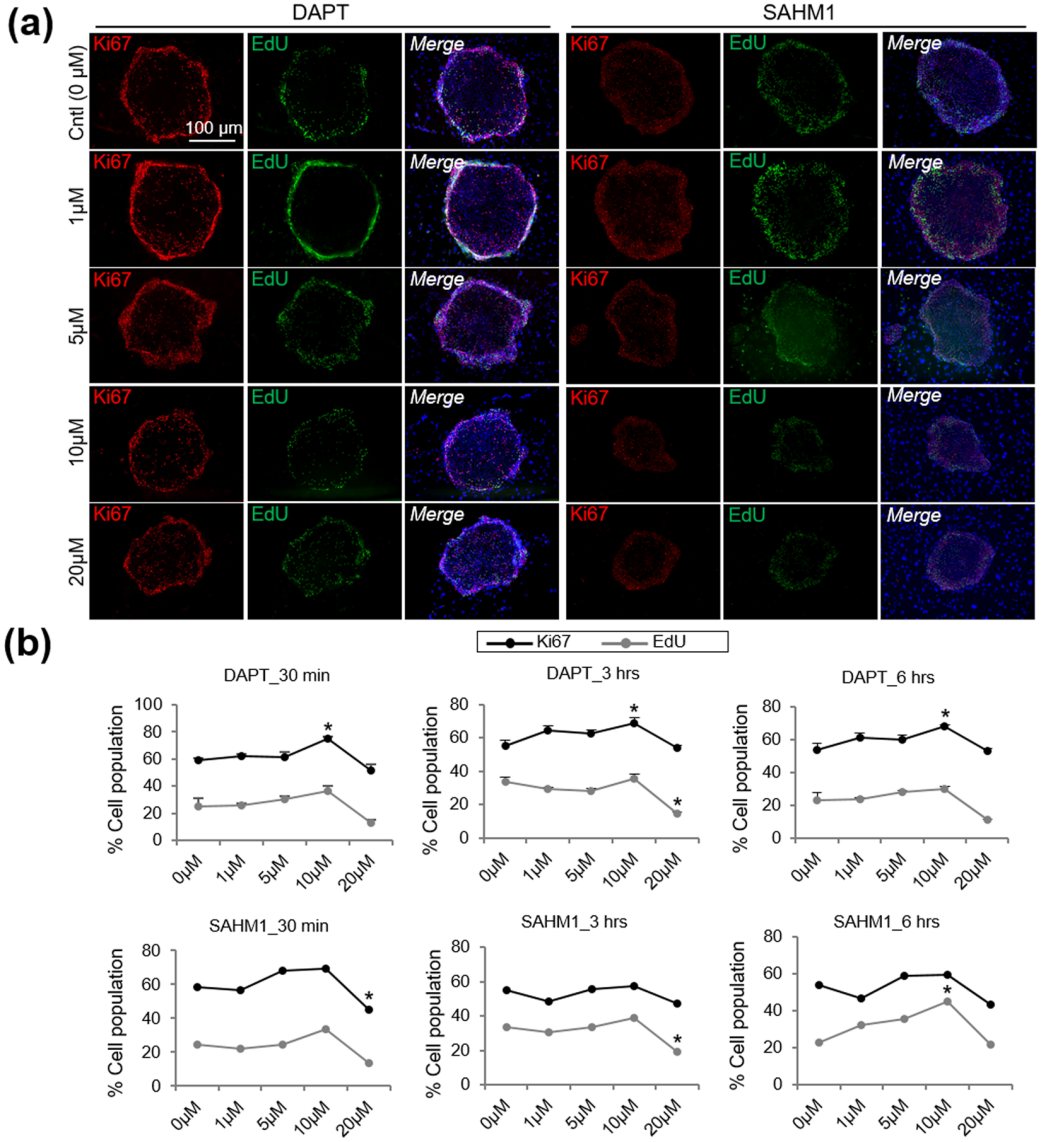
Ki67 and EdU expression and localization in the LSC colonies after incubation with DAPT/SAHM1 at different concentrations. **(a)** Representative images of EdU and Ki67 in the LSC colonies after a 30-minute incubation with DAPT/SAHM1 at different concentrations. **(b)** Quantification of the Ki67^+^ and EdU^+^ cell population in the entire colony after a 30-minute, 3-hours and 6-hours incubation with DAPT/SAHM1 at different concentrations. Data are represented as mean ± SEM. See also Fig. S1. Abbreviations: Cntl, control; LSC, limbal stem/progenitor cells.

The strongest Ki67 expression was localized at the periphery of the colonies (Fig. 5A). Control LSC cultures or cultures with a low DAPT/SAHM1 concentration had the lowest expression of Ki67 at the center of the colony (Fig. 5A). This is consistent with cell morphology and colony size results obtained. Instead, smaller colonies found when incubating with high concentrations of DAPT/SAHM1 showed a more homogenous Ki67 expression within the colony (Fig. 5A). We observed a significant increase in the percentage of Ki67^+^ cells in the entire colony only for 10 μM DAPT at all time points (*P* = 0.0003, *P* = 0.01 and *P* = 0.02, respectively; Fig. 5B). SAHM1 at 20 μM after 30 minutes significantly decreased the number of Ki67^+^ cells (*P* = 0.03; Fig. 5B).

EdU incorporation (Fig. 5B) was significantly increased for 10 μM of SAHM1 after 6 hours (*P* = 0.008). DAPT and SAHM1 at 20 μM after 3 hours incubation led to a reduction in the number of EdU^+^ cells (*P* < 0.05).

EdU incorporation at the periphery of the colony (Supplementary Fig. S1) after 30 minutes of incubation with DAPT or SAHM1 is lower compared to that in the control (*P* < 0.05; Supplementary Fig. S1). However, after 3 and 6 hours of the incubation with DAPT or SAHM1, EdU incorporation in the DAPT and SAHM1 groups significantly increased compared to the control at most of the concentrations tested (*P* < 0.05; Supplementary Fig. S1).

In the center of the colony, EdU incorporation after 30 minutes showed the opposite trend as in the periphery of the colony (Supplementary Fig. S1). After 30 minutes of incubation with either DAPT or SAHM1, the percentage of EdU^+^ cells increased in high concentrations up to 10 µM (Supplementary Fig. S1). After 3 and 6 hours though, the trend was similar to the one found at the periphery; however, the overall amount of EdU^+^ cells in the center of the colonies were lower than at the periphery (Supplementary Fig. S1). After 6 hours of DAPT/SAHM-treatment, the percentage of EdU^+^ cells was similar among all the conditions (Supplementary Fig. S1).

The percentage of Ki67^+^ cells found at the peripheral and central areas of the colonies (Supplementary Fig. S1) was similar to the percentage of Ki67^+^ cells analyzed on the single harvested cells (Fig. 3E). This demonstrates that the absence of Ki67 expression in the center of the colony is not artifact due to a lower penetration of the antibody in the center of the colonies.

### Notch inhibition did not reduce the ability of LSCs to stratify

**Figure 6:**
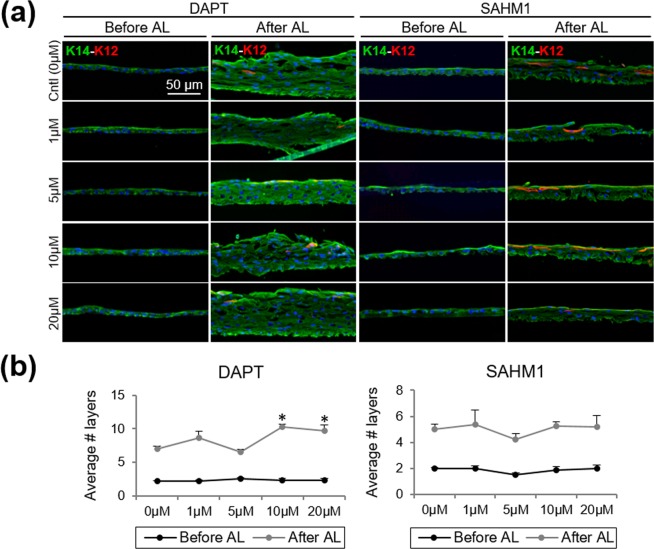
Stratification and differentiation of the LSC sheets in the presence of DAPT and SAHM1. **(a)** Representative images of double K14-K12 staining using different concentrations of DAPT/SAHM1 before and after air-lifting (AL) induction. **(b)** Quantification of the number of layers (stratification) in the presence of DAPT/SAHM1 before and after AL. Data are represented as mean ± SEM. Abbreviations: AL, air-lifting; K12, cytokeratin 12; K14, cytokeratin 14; LSC, limbal stem/progenitor cells.

Air-lifting promotes stratification and differentiation of the corneal epithelial cells. A control group from the same donor was always included to control for the variation from the donor tissues. Before air-lifting, no significant differences were found in the average number of layers between LSC cultures in the presence of DAPT or SAHM1 and control groups (*P* > 0.05; Fig. 6A,B). After air-lifting, stratification occurred in both the control and DAPT/SAHM1-treated cultures (Fig. 6A,B). DAPT at high concentrations showed an increased stratification capacity with regards to the control (10 μM DAPT, *P* = 0.007, and 20 μM DAPT, *P* = 0.04; Fig. 6B). Absence or minimal K12 expression was observed in the submerged LSC cultures before air-lifting and an increased K12 expression was observed after air-lifting in all conditions tested (Fig. 6A, Fig. S2).

The percentage of the p63α^bright^ stem/progenitor cells significantly increased in the presence of DAPT at 20 μM (*P* = 0.009; Supplementary Fig. S2). The percentage of p63α^bright^ cells at the basal layer remained similar in all other conditions tested.

## Material and Methods

### Limbal stem cell culture

Human sclerocorneal tissues from 20- to 70-year-old healthy donors were obtained from different eye banks. Human tissue was handled in accordance with the tenets of the Declaration of Helsinki. The experimental protocol was exempted by the University of California Los Angeles Institutional Review Board (IRB#12-000363). The death-to-preservation time interval was 4–16 hours (mean 8.7 ± 1.2 hours). The death-to-experiment time interval was 4–7 days (mean 5.9 ± 0.4 days).

Limbal stem cell cultures were performed as previously described^19–22^. Briefly, after incubation in a 2.4 U/mL of dispase II (Roche; Indianapolis, IN) solution at 37 °C for 2 hours in DMEM/F-12 medium (Life Technologies; Carlsbad, CA), limbal epithelial cell sheets were then isolated from the slcerocorneal tissue and subjected to digestion with 0.25% trypsin-1 mM EDTA (Life Technologies) for 5 minutes. Single limbal epithelial cells were seeded at a density of 300 cells/cm^2^ on sub-confluent 3T3-J2 mouse fibroblasts (3T3; the Howard Green laboratory, Harvard Medical School) that had been growth-arrested using 4 µg/mL of mitomycin C (Sigma-Aldrich; St. Louis, MO) for 2 hours at 37 °C.

LSCs were cultured using supplemental hormone epithelial medium (SHEM) that consisted of DMEM/F12 medium supplemented with 5% fetal bovine serum (FBS; Life Technologies), N2 supplement (Life Technologies), 2 ng/mL of epidermal growth factor (EGF; Life Technologies), 8.4 ng/mL of cholera toxin (Sigma-Aldrich; St. Louis, MO), 0.5 µg/mL of hydrocortisone (Sigma-Aldrich), 0.5% of dimethyl sulfoxide (DMSO; Sigma-Aldrich), penicillin-streptomycin (Life Technologies) and gentamicin/amphotericin B (Life Technologies). DAPT (Sigma-Aldrich) and SAHM1 (EMD Millipore; Billerica, MA) were added to the culture medium at the concentration of 1, 5, 10 and 20 µM. To compensate for the DMSO effect in the different concentrations of small molecules added, the final concentration of DMSO was adjusted for all cultures to be 0.5% (vol/vol). Medium was changed every 2 days.

Images of cell cultures were taken with an inverted DMIL LED microscope (Leica Microsystems, Wetzlar, Germany). Cell size was measured on 10X images of the harvested cells by using ImageJ software (US National Institutes of Health, Bethesda, MD). The percentage of cells ≤12 µm was calculated for each culture condition.

Notch inhibition by DAPT and SAHM1 was tested in fresh isolated limbal epithelial cells. LN rat cells expressing N1 ectopically (LN1-7 cells) were used as a positive control^18^. LN1-7 cells were cultured in DMEM (Life Technologies) supplemented with 10% FBS.

### Colony forming efficiency (CFE)

LSC cultures were fixed with 3.7% formaldehyde (Sigma-Aldrich) for 10 minutes and stained with 0.5% rhodamine B (Sigma-Aldrich) for 15 minutes to visualize the colonies. CFE was obtained by dividing the number of colonies by the number of cells seeded.

### Air-lifting induction

Air-lifting is a common maneuver to induce stratification and differentiation that was first described in mouse^23^ and human^24^ skin keratinocytes. Air-lifting was induced in the LSC cultures by lowering the amount of culture medium to expose the cells to the air-liquid interface in the absence of DAPT or SAHM1 small molecules. Air-lifted LSC cultures were then fixed in 4% paraformaldehyde (PFA; Thermo Fisher Scientific, Ward Hill, MA), embedded in optimal cutting temperature (OCT) solution (Thermo Fisher Scientific) and stored at −80 °C. Tissue sections of 8 µm thickness were obtained by using the CM3050 S Cryostat (Leica; Buffalo Grove, IL). The amount of stratification in the different culture conditions was measured by quantifying the number of cell layers after K14 immunostaining. Differentiation was revealed by positive expression of K12 on the culture sections.

### Western blot

The cultivated LSCs were lysed using RIPA buffer (Sigma-Aldrich) supplemented with protease-phosphate inhibitor cocktail (Cell Signaling Technology, Danvers, MA). Protein concentration was determined using the Micro BCA protein assay kit (Thermo Fisher Scientific). Lysates containing 10 µg of total protein content were separated using one-dimensional NuPAGE 4–12% Bis-Tris gel (Thermo Fisher Scientific) and transferred to polyvinylidine difluoride membranes. Blocking was done using SuperBlock blocking buffer (Thermo Fisher Scientific) followed by immunoblotting overnight at 4 °C using the primary antibodies (Supplementary Table S1), and HRP-conjugated secondary antibodies for 2 hours at room temperature. The chemiluminescent kit SuperSignal West Dura Extended Duration Substrate (Thermo Fisher Scientific) was used to develop the blot.

### Immunohistochemistry

Immunostaining was performed on cytospin slides, whole mounts and cryosections from both human sclerocorneal tissue and cell culture sections. Cytospin slides were prepared using the cytocentrifuged (Cytofuge; Thermo Scientific, Waltham, MA) and stored at −20 °C. Whole mounts were immunostained right after the culture period. Fixation was done using 4% PFA followed by blocking and permeabilization in PBS containing 1% bovine serum albumin (Sigma-Aldrich) and 0.5% Triton X-100 (Sigma-Aldrich) for 30 minutes. Incubation with primary antibodies was done overnight at 4 °C (Supplementary Table S1) followed by incubation with secondary antibodies for 1 hour at room temperature. Nuclei were counterstained with Hoechst 33342 (Life Technologies). Images were acquired by using a Zeiss Image.A2 fluorescent microscope (Carl Zeiss Inc., Oberkochen, Germany) or a confocal microscope (Confocal Laser Scanning Microscopy; Olympus, San Jose, CA).

Quantification of cells expressing high levels of p63α (p63α^bright^ cells) was done using the Definiens Tissue studio software (Larchmont, NY) as previously reported^25^. Quantification of cytokeratin K14, K12 and Ki67 expression was done using ImageJ software.

### Quantitative RT-PCR

Total RNA was extracted by using RNeasy Mini Kit (Qiagen) and underwent DNase treatment (Ambion Inc, Austin, TX). The quantity and quality of total RNA were assessed using a spectrophotometer (NanoDrop 1000; NanoDrop, Wilmington, DE). Reverse-transcription was performed using the Superscript II RNase H2 reverse transcriptase kit (Invitrogen). The relative abundance of transcripts was detected by quantitative (q) RT-PCR (KAPA SYBR FAST qPCR Master Mix; Stratagene, La Jolla, CA). The housekeeping gene glyceraldehyde-3-phosphate dehydrogenase (GAPDH) was used to normalize the fluorescence level. The primers used for qRT-PCR are listed in Supplementary Table S2.

### EdU incorporation assay

Serum starvation was performed overnight to synchronize the LSC cultures. The ability of the cells to proliferate was measured by using the EdU incorporation assay Click-iT Plus EdU Imaging Kit (Molecular Probes, Eugene, OR) according to the manufacturer’s recommendations, after a 30-minute, 3-hour and 6-hour incubation with DAPT or SAHM1. A 30-minute incubation with 10 µM EdU was performed followed by fixation with 4% PFA.

### Statistical analysis

Data were statistically analyzed by using the pairwise t-test from at least 4 independent experiments using 4 different donor tissues. Graph bars are expressed as the mean ± standard error of the mean (SEM). Values with a *P* < 0.05 were considered as statistically significant.

## Discussion

We have confirmed the expression of some key members involved in the Notch signaling pathway in the human corneal and limbal epithelium including the receptors N1, N2 and the target genes HES1 and HEY1. This is consistent with previous observations from other studies in human^9–13^, mouse^11^ and rat^26^. Differences in the expression patter of the Notch members may result from the different species analyzed. The N1 receptor was strongly expressed at the basal layer of the limbus where the LSCs reside. Notch signaling activation given by the expression of N1IC, HES1 and HEY1 *in vivo* was only observed in a few of the adult tissues stained. Most of them showed expression of the N1 receptor at the membrane and HES1/HEY1 had a cytoplasmic-perinuclear distribution.

In the LSC niche, other than the receptor-ligand interaction from opposing cells (trans-interaction), ligand and receptor could autonomously interact (cis-interaction) in the same cell promoting Notch inhibition^27–29^. The regulation of Notch signaling at the basal layer of the limbus is still poorly understood. These results suggest that in adult tissue, Notch activation may occur sporadically, when regeneration of the corneal surface is needed during normal homeostasis or wound healing.

The results from the Notch inhibition in the LECs suggest the involvement of Notch signaling in the regulation of the limbal epithelium. Notch activation occurred after the LECs were isolated and cultured. DAPT and SAHM1 were able to inhibit Notch signaling in the LECs. Short-term inhibition in the LSCs showed decreased expression of the target genes HES1/5 and HEY1 at the mRNA level and nuclear N1IC at the protein level. The inhibition of Notch signaling in the LSCs by DAPT has been shown before^9,26^. However, we report for the first time the inhibition by SAHM1 in the LSCs. SAHM1 has been previously used to inhibit Notch signaling in human breast cancer cells^30^, leukemic cells^17^ and osteoclast precursors^31,32^.

SAHM1 is a cell-permeable peptide that specifically prevents the assembly in the Notch transcription complex. DAPT is a broad inhibitor that targets gamma secretase, an integral membrane protein that cleaves type I transmembrane proteins such as Notch^33^, ErbB4^34^, E-cadherin^35^, N-cadherin^36^, ephrin-B2^37^ and CD44^38^.

In our study, DAPT promoted a stronger inhibition than did SAHM1. Consistent with our results, it has been showed that interacting upstream with the enzyme is easier than with a specific protein downstream in the signaling pathway^39^. Protein-protein interactions are difficult to block with small molecule inhibitors; however, enzymes that catalyze certain chemical reactions like gamma secretase are easier to inhibit. Also, it has been shown that the production of downstream targets is rapidly increased in response to the presence of the inhibitor; however, if the enzyme upstream is blocked, the production of the target downstream will be inhibited by using a lower concentration of the drug^40^. By using a more specific inhibitor like SAHM1, we are specifically targeting a desired signaling pathway; however, by using a broad spectrum inhibitor like DAPT, we might be blocking some other signaling pathways that might be important for normal functioning^40^. A balance between the desired target effect and appropriate concentration of the drug to avoid toxicity has to be determined in drug therapy.

The effect of Notch inhibition on the LSC phenotype was consistent using both small molecules. We found a trend of increasing the number of small cells (≤12 µm), and decreasing the percentage of differentiated K12^+^ cells when Notch signaling is inhibited. The compact LSC-like morphology, CFE, percentage of K14^+^ cells and p63^bright^ cells was maintained in all cultures at all concentrations of the small molecules tested.

Proliferation in the LSC cultures treated with the Notch inhibitors seems to decrease specially at high concentrations of both DAPT and SAHM1. After serum starvation, the LSCs were synchronized at G0. Upon reentering the cell cycle by adding serum in the medium, proliferation at the periphery of the colony starts earlier in the control group compared to the DAPT/SAHM1-treated groups. However, in the center of the colony we found the opposite trend. In smaller colonies, the closer proximity of the LSCs from the center with the feeders might be promoting a higher proliferation in the DAPT/SAHM1-treated groups. The expression pattern of EdU seems to indicate that there is a delay in entering S phase in the DAPT-SAHM1-treated cultures. In addition, regional differences observed in the colonies could be due to differences in the regenerative potential of the cells. Cells in the center of big colonies which are further away from the trophic factors of the feeder cells are more differentiated as we previously shown^41^, thus had a lower proliferation capacity.

The decrease in the proliferation rate after Notch inhibition *in vitro* could be due to differences in the phases of the cell cycle. Notch signaling is required for the normal post-mitotic differentiation of multiple epithelial stem cell systems^42,43^. Cell cycle progression and cell fate are linked. A quiescent slow cycling phenotype is correlated with a stem cell phenotype *in vivo*. It has been shown that slow cycling hematopoietic stem cells have the highest long-term stem cell potential^44^. Moreover, the level of Notch signaling activation has been directly implicated in the maintenance of quiescence in several stem cell systems such as muscle stem cells^45^, pancreatic endocrine progenitors^46^ and neural stem cells^47^.

The role of Notch signaling in the differentiation of the epidermal keratinocytes has been investigated before^48^. In the eye, Notch signaling also promotes differentiation in the epithelium of the Meibomian gland^49^. Corneal epithelium differentiation and wound healing has been shown to be regulated by Notch signaling^11,14^. Particularly, Notch inhibition has been shown to reduce the amount of proliferating cells in human LSC cultures^9^, and a more recent study found that rat LSC cultures have a lower differentiation and proliferation rate after Notch inhibition^26^. Moreover, the role of Notch1 in epithelial differentiation and recovery after wound healing have been proposed as key factors for the maintenance of a good corneal epithelial barrier function^15^.

Other studies have suggested that Notch signaling suppresses differentiation in a rat corneal wound healing model without affecting proliferation^50^. On the other hand, Notch activation has been shown to induce a rapid proliferation after wounding^14^ in a corneal wound healing mouse model. Differences among these studies and our results could be due to the species analyzed and to the different effect of Notch signaling during wound healing.

The ability of the LECs to stratify and differentiate after Notch inhibition was shown through the air-lifting experiments. Air-lifting technique is used to generate epithelial cell sheets with barrier functions, controlled proliferative activity in the basal cells and differentiated superficial cells. Basal stem/progenitor cells retaining cell polarity are essential to induce stratification and differentiation at the superficial layer of the epithelium. Air-lifting has proven to reduce intercellular spaces in the superficial cells and promotes the formation of the barrier function^51^. Moveover, air-lifting has been shown to increases stratification, enlarge surface cells, triggers cellular differentiation and increases transepithelial electrical resistance, while  maintaining the stem/progenitor cell properties at the basal layer^52^. By contrast, air-lifting was proposed to induce epithelial to mesenchymal transition (EMT) of LSCs and instrastromal invasion^53^ when cultured with epithelial-stromal tissue recombinants.

Upon air-lifting, we observed that control LECs were able to stratify and differentiated while retaining the stem/progenitor cell population at the basal layer, given by the expression of p63α^bright^ cells. After Notch inhibition by DAPT and SAHM1, the ability of the LECs to stratify and differentiate was not reduced. LECs were able to reproduce a stratified limbal epithelium with several layers and differentiated K12^+^ cells at the surface and high p63α expression at the basal layer that was comparable to the control LECs. P63 has been shown to regulate the molecular switch for initiating the epithelial stratification and maintaining the proliferation of basal keratinocytes in the mature epidermis^54^. This is an indication that Notch inhibition does not impair the capacity of the LSCs to stratify and differentiate.

Differences observed in the stratification between DAPT and SAHM1 cultures could be due to the fact that DAPT is broad gamma-secretase inhibitor that affects several signaling pathways other than Notch. Gamma-secretase cleavage releases the intracellular domain of Cadherins and regulates disassembly of adherens junctions, which in turns increases cytoplasmic amount of beta-catenin available for initiating Wnt signaling^35^. In the presence of DAPT, adherens junctions could be preserved, which in turn might decrease cell proliferation and maintain the barrier function. Once DAPT is removed and air-lifting is induced, we observed an increase in the number of cell layers (stratification) that was more pronounced than that observed in the presence of SAHM1. This could be because the undifferentiated phenotype of the cell population at the basal layer is better preserved in the presence of DAPT.

In conclusion, manipulation of Notch signaling in the LSCs can be achieved by using small molecules. Inhibition of Notch signaling decreases the proliferation of LSCs but preserves the undifferentiated phenotype. Further research is needed to further elucidate the mechanism of the Notch inhibition in the LSCs.

## Supplementary information

Supplementary Information

## Data Availability

All data generated or analysed during this study are included in this published article (and its Supplementary Information files).
