# Supplementary material for: Notch Inhibition Prevents Differentiation of Human Limbal Stem/Progenitor Cells in vitro
Source: Sci Rep. 2019 Jul 17;9:10373. doi: 10.1038/s41598-019-46793-6 (PMC6637172; doi:10.1038/s41598-019-46793-6)
Supplement: Supplementary file 1 — Supplementary Information [file 41598_2019_46793_MOESM1_ESM.pdf]

Notch Inhibition Prevents Differentiation of Human Limbal Stem/Progenitor Cells *in vitro*

Sheyla González[1], Heui Uhm[2] and Sophie X. Deng[1*]

[1]Cornea Division, Stein Eye Institute, University of California, Los Angeles, CA 90095, USA.

[2]David Geffen School of Medicine, University of California, Los Angeles, CA 90095, USA.

***Corresponding author**:
Sophie X. Deng, M.D., Ph.D.
Professor of Ophthalmology
Stein Eye Institute, University of California, Los Angeles
100 Stein Plaza
Los Angeles, CA 90095
Tel: 310-206-7202
Fax: 310-794-7906
E-mail: deng@jsei.ucla.edu

Supplementary Tables

Supplementary Table S1. Primary antibodies used for immunocytochemistry, immunohistochemistry and western blot.

Primary Antibody	Dilution	Source and Catalogue #
K12	1:100	Santa Cruz Biotechnology sc-25722
K14	1:50	Fisher Scientific MS-115-R7
p63α	1:100	Cell Signaling Technology #4892
Notch1	1:100	R&D Systems AF3647
Cleaved Notch1	1:100	Cell Signaling Technology #4147
GAPDH	1:100	EMD Millipore MAB374
HES1	1:100	Abcam ab71559
HEY1	1:100	Abcam ab22614
Ki67	1:100	DAKO M7240

Abbreviations: GAPDH, glyceraldehyde-3-phosphate dehydrogenase; HES1, hairy and enhancer of split-1; HEY1, hairy/enhancer-of-split related with YRPW motif protein 1; K12, cytokeratin 12; K14, cytokeratin 14.

Supplementary Table S2. Primers used for qRT-PCR.

Gene	Direction	Primer Sequence (5'→3')	GeneBank Accession number
GAPDH	Forward	CGACCACTTTGTCAAGCTCA	NG_007073.2
	Reverse	AGGGGTCTACATGGCAACTG	
ABCG2	Forward	CCGCGACAGCTTCCAATGACCT	NG_032067.2
	Reverse	GCCGAAGAGCTGCTGAGAACTGTA	
ΔNp63	Forward	TCCATGGATGATCTGGCAAGT	NG_007550.1
	Reverse	GCCCTTCCAGATCGCATGT	
N-cadherin	Forward	GAGGAGTCAGTGAAGGAGTCA	NG_011959.1
	Reverse	GGCAAGTTGATTGGAGGGATG	
K14	Forward	GACCATTGAGGACCTGAGGA	NG_008624.1
	Reverse	ATTGATGTCGGCTTCCACAC	
K12	Forward	CCAGGTGAGGTCAGCGTAGAA	NG_008077.1
	Reverse	CCTCCAGGTTGCTGATGAGC	
Ki67	Forward	CTTTGGGTGCGACTTGACG	NC_000010.11
	Reverse	GTCGACCCCGCTCCTTTT	
Snail1	Forward	CAGCTATTTCAGCCTCCTGTT	NC_000020.11
	Reverse	CCGACAAGTGACAGCCATTA	
Twist	Forward	TCACACCTCTGCATTCTGATAG	NG_008114.1
	Reverse	CTGATTGGCACGACCTCTT	
Vimentin	Forward	CAGCTTTCAAGTGCCTTTCTG	NG_012413.1
	Reverse	CTTGTAGGAGTGTCGGTTGTT	

Abbreviations: ABCG2, ATP-binding cassette sub-family G member 2; GAPDH, glyceraldehyde-3-phosphate dehydrogenase; K12, cytokeratin 12; K14, cytokeratin 14.

Supplementary Figures

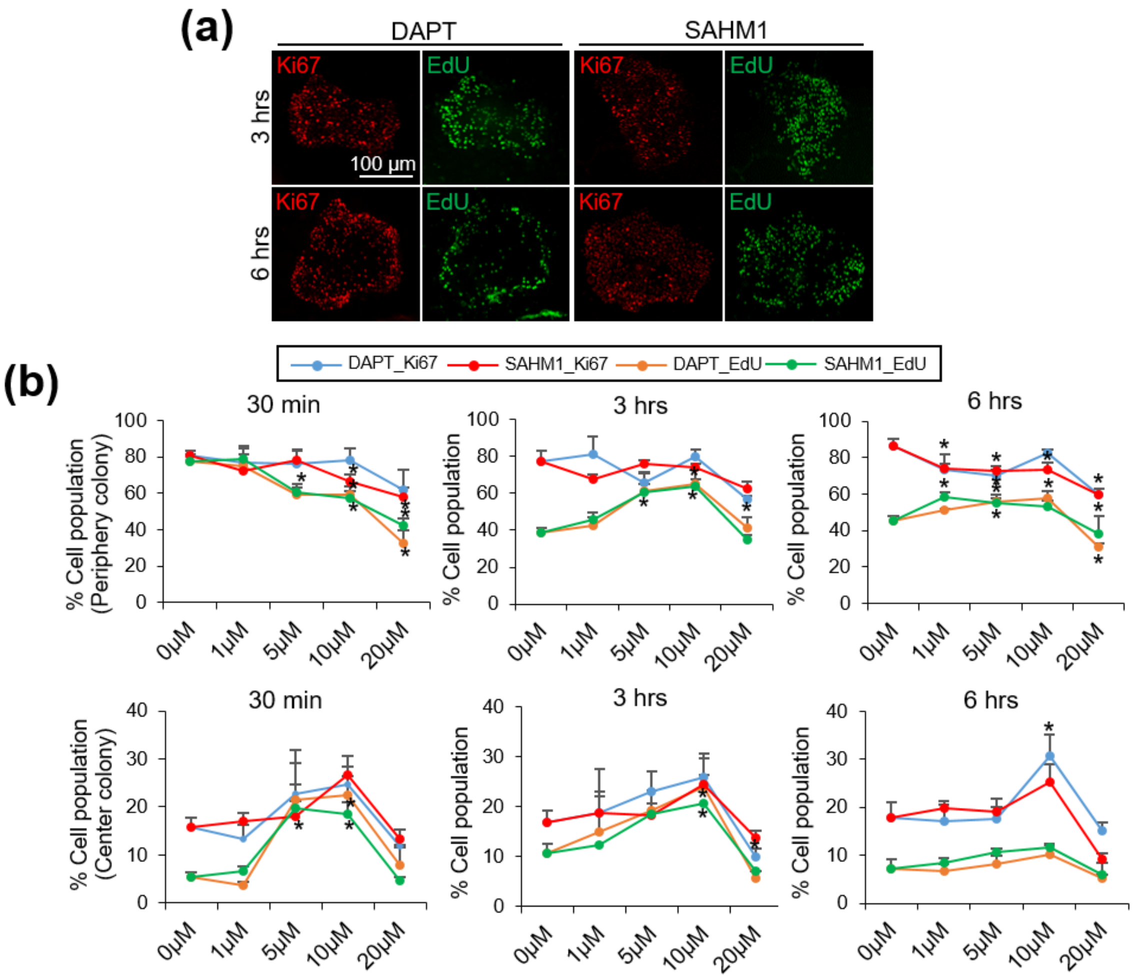

Supplementary Fig. S1. Ki67 and EdU expression and localization in the LSC colonies after a 30-minute, 3- and 6-hours incubation with DAPT/SAHM1. **(a)** Representative images of EdU and Ki67 in the LSC colonies. **(b)** Quantification of the Ki67⁺ and EdU⁺ cell population in the center and periphery of the colony after a 30-minute, 3-hours and 6-hours incubation with DAPT/SAHM1 at different concentrations.

Data are represented as mean ± SEM.

Abbreviations: LSC, limbal stem/progenitor cells.

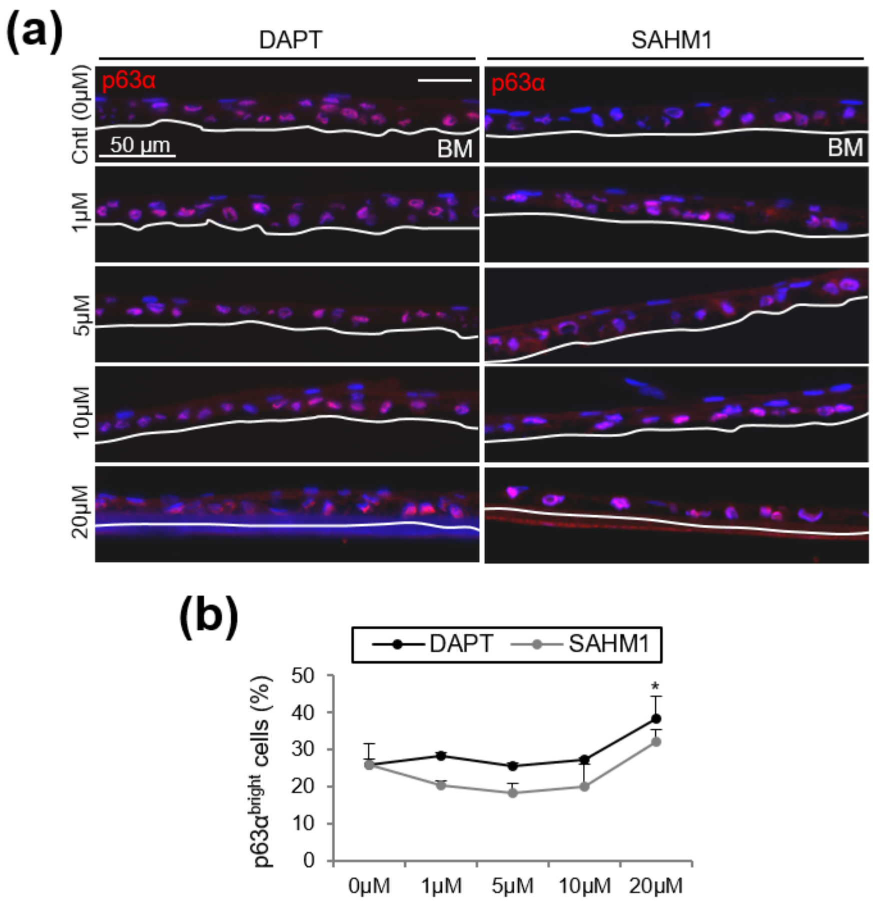

Supplementary Fig. S2. Quantification of p63α^{bright} cells at the basal layer of the LSC cultures with DAPT/SAHM1 at different concentrations. **(a)** Representative pictures of p63α on cross-sections of LSC cultures. **(b)** Quantification of p63α^{bright} cells at the basal layer.

Data are represented as mean ± SEM.

Abbreviations: BM, basement membrane; Cntl, control; LSC, limbal stem/progenitor cells.

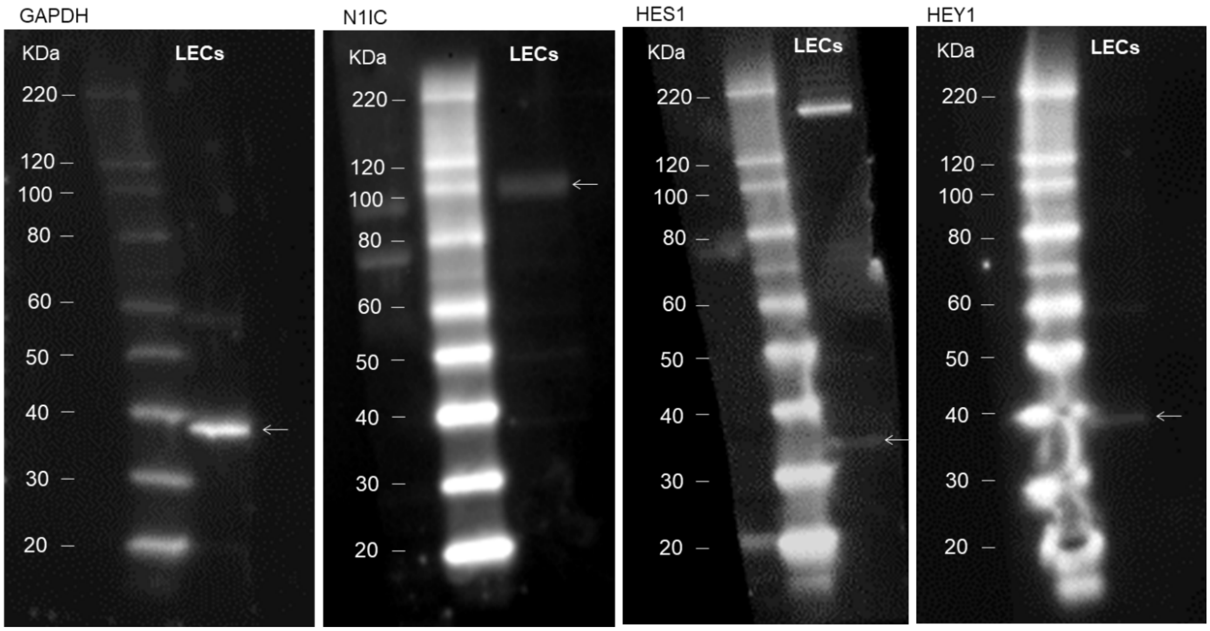

Supplementary Fig. S3. Uncropped images of blots shown in Figure 1 for GAPDH, N1IC, HES1 and HEY1.

Abbreviations: GAPDH, Glyceraldehyde-3-Phosphate Dehydrogenase; HES1, hairy and enhancer of split-1; HEY1, hairy/enhancer-of-split related with YRPW motif protein 1; N1IC, Notch1 intracellular domain.
